# Responsible Research and Innovation as a Novel Approach to Guide Educational Impact of Mind, Brain, and Education Research

**DOI:** 10.1111/mbe.12213

**Published:** 2019-09-29

**Authors:** Nienke van Atteveldt, Geertje Tijsma, Tieme Janssen, Frank Kupper

**Affiliations:** ^1^ Faculty of Behavioural and Movement Sciences, Section of Clinical Developmental Psychology and Institute Learn! Vrije Universiteit Amsterdam; ^2^ Faculty of Beta Sciences, Athena Institute Vrije Universiteit Amsterdam

## Abstract

We propose a Responsible Research and Innovation (RRI) framework to improve the alignment between mind, brain, and education (MBE) research, the educational practice, and other societal stakeholders. RRI is an approach that has successfully been used in different research fields, but not yet in MBE research. After substantiating the need for, and possibilities of using this framework within MBE research, we report a case study to demonstrate the feasibility and benefits of RRI within an MBE context. This case study entails developing an educational intervention to improve learners' sense of agency regarding their own learning processes using neurofeedback. Using RRI, we found that societal stakeholders (teenagers, parents, and teachers) anticipate different potential impacts of this neurotechnology‐based intervention than researchers did, enabling us to adapt the intervention according to these perspectives. This example demonstrates that RRI enables researchers to be reflexive and responsive to the stakeholders needs and values, to ultimately improve the educational and societal value of MBE research.

Cognitive neuroscience research has increased our understanding of brain development and learning significantly. In pursuing the aim of using these insights to improve learning and teaching in practice, a new field has emerged, either called educational neuroscience or mind, brain, and education (MBE) (Ansari, Coch, & De Smedt, 2011). However, the direct educational value of neurobiological insights in for example attention, reading acquisition or memory is limited by several challenges, including miscommunications (Howard‐Jones, 2014) and the low ecological validity of standard cognitive neuroscience studies (Kingstone, Smilek, & Eastwood, 2008; Matusz, Dikker, Huth, & Perrodin, 2019; van Atteveldt, van Kesteren, Braams, & Krabbendam, 2018). In recent review and opinion papers (e.g., Gabrieli, 2016; Howard‐Jones, 2014; Varma, McCandliss, & Schwartz, 2008), many researchers call for a better collaboration with the educational practice, to avoid misconceptions and to improve the match between the scientific research and the needs in the practice. The link between neuroscience and education needs to be a two‐way dialogue (Ansari et al., 2011).

Also from a science communication perspective it has been proclaimed that public engagement and dialogue is needed especially in neuroscience research, to avoid misinterpretation and to discuss emerging ethical and social issues (Illes et al., 2010; O'Connor, Rees, & Joffe, 2012; van Atteveldt, van Aalderen‐Smeets, Jacobi, & Ruigrok, 2014). In addition, previous work has shown that more and better dissemination by researchers may not be as effective as more active collaborations between researchers and education professionals (Edelenbosch, Kupper, Krabbendam, & Broerse, 2015). Although there are quite some examples of successful teacher‐researcher collaborations (e.g., McCandliss, Kalchman, & Bryant, 2003), a *systematic* approach for how to actively include the educational practice *during all stages* of MBE research is lacking. As it takes considerable effort and time for individual researchers to fulfill the need for dissemination and public dialogue, efficient and accessible approaches are much needed (Illes et al., 2010).

In this article, we put forward the Responsible Research and Innovation (RRI) framework, originating at the intersection of technology assessment, ethics and science policy, as a highly adequate systematic approach to involve the educational practice and other societal stakeholders in MBE research. The RRI framework integrates ethical reflection, public engagement, and responsive change (Stilgoe, Owen, & Macnaghten, 2013). One of the prime characteristics is the shift in focus from managing risks to the question of right impacts: What is the contribution we want science and technology to make? Another important characteristic is the shift from individual responsibility to collective responsibility. RRI envisions a process of sharing responsibility by means of the active and early participation of a wide range of actors in integrated processes of inclusive deliberation coupled to policy‐making and action (Owen, Macnaghten, & Stilgoe, 2012).

Over the past years, some studies used certain elements of RRI in the context of MBE research. For example, Edelenbosch et al. held focus groups with different stakeholder groups to reflect on opportunities and concerns of applying neuroimaging to individualized learning (Edelenbosch, Kupper, & Broerse, 2015). Their study yielded insights to optimize framing of future research with such applications as goal. Other studies focused on ethical issues, for example, Howard‐Jones and Fenton (2012) gathered survey data to investigate educator's opinions on the ethics of cognitive enhancement and other developments. This study showed the need for interdisciplinary and public discussion of neurocognitive ethical issues. Other studies included stakeholder inputs at some stage during the design process, for example, resulting in interventions with optimal learner engagement (e.g., Wilson et al., 2006). In sum, previous studies involving societal stakeholders in MBE research have yielded interesting results regarding specific aspect of societal or educational impact (e.g., framing of communication, ethics, or designing ecologically valid materials). However, we are not aware of any study that has integrated RRI or a similar framework from the start to the end of the research, to cover all the ensuing issues together.

In our view, a more comprehensive approach to involve stakeholders is needed, given the large variety of societal fields MBE research has an impact on. RRI provides researchers with the opportunity to deliberate with societal stakeholders on impacts, such as who benefits from these new developments and what are the potential risks. Moreover, applying RRI to MBE research has the potential to increase the educational value of the research. First, the practical and experiential knowledge of stakeholders may improve the focus and direction of research aiming to make a difference in educational practice (Escobar, 2013), as well as increase its ecological validity (van Atteveldt et al., 2018). Second, including society in an upstream dialogue may avoid misunderstanding of MBE research. This is important, as miscommunication about how the brain works or about the meaning of neuroscientific findings is an important hurdle in translating MBE findings to the practice (Howard‐Jones, 2014). Finally, from a democratic perspective, societal stakeholders have the *right* to be included when considering innovations that might have an impact on them (Kupper, Klaassen, Rijnen, Vermeulen, & Broerse, 2014). In the following, we will first describe the background and dimensions of the RRI approach and next, we will illustrate how RRI can be applied to MBE research by means of a case study focusing on the responsible implementation of a neurofeedback intervention aimed to make students aware of the plasticity of their own brain and to improve their sense of agency regarding their own learning processes.

## ORIGINS, DIMENSIONS, AND EXAMPLES OF THE RRI APPROACH

New scientific and technological developments, such as research on the implementation of neurotechnology in classrooms, are commonly praised for their potential to generate numerous benefits, but at the same time accompanied by issues concerning the distribution of benefits and risks, and potential impacts on the lives of people and society (see for a critical review Williamson, 2019). The question is how to deal with these issues. Science and technology studies have long emphasized that the artifacts of science and technology are not only technically, but also socially and politically constructed (see Latour & Woolgar, 1979). The growing recognition that technology and society mutually shape each other has led to the introduction of various platforms to influence reflection and decision‐making processes in order to steer the development of technologies toward more acceptable ends. Recently, the RRI framework emerged as a form of anticipatory governance, aimed at modulating research and innovation trajectories toward the right impacts (Collingridge, 1980; Gibbons, 1999; Owen et al., 2012). In a few years, the term has gained increasing relevance in the governance of science and technology in Europe. Various conceptualizations of RRI have emerged in scientific and policy literature. Common features involve (1) a focus on societal challenges, (2) active engagement of a range of stakeholders, (3) anticipating problems, solutions, and alternatives and reflecting on underlying values and assumptions, (4) a willingness to be responsive, act, and adapt (Stilgoe et al., 2013; Von Schomberg, 2013; Wickson & Carew, 2014).

MBE research is dealing with numerous developments that might impact society and should therefore be aligned with the values, needs, and interests from the societal stakeholders. To effectuate this alignment, we propose the RRI framework developed by Stilgoe et al. (2013), which has likely been the most influential conceptualization around. It consists of four dimensions: anticipation, reflection, inclusion, and responsiveness (see Table 1). *Anticipation* is required to understand how the dynamics of Research & Innovation (R&I) shape the future. Detrimental effects are often unforeseen, signifying the importance of envisioning the impacts of dominant and alternative R&I futures. Anticipation should go hand in hand with *reflexivity* on the part of both actors and institutions. Reflexivity is conceived as holding a mirror to one's own activities, commitments, and underlying perspectives on problem definitions and preferred solutions. The dimension of *inclusion* represents the early involvement of a wide range of stakeholders in deliberation and decision‐making processes, both for normative democratic reasons and to broaden and diversify sources of expertise, disciplines, and perspectives. Finally, responsible R&I requires that the anticipatory and reflective questions generated in inclusive deliberations are also responded to. The dimension of *responsiveness* refers to the development of a capacity to change existing routines of thought and behavior, as well as overarching organizational structures and systems.

**Table 1:** Overview of the Responsible Research and Innovation (RRI) Dimensions, Implementation Strategies in the Current Case Study, and the Main Findings

RRI dimension	Clarification	Implementation in current case study	Summary of results
Reflexivity	Reflecting on one's own activities and perspectives on problem definitions and preferred solutions	Reflecting on the stakeholders' vision of the brain and its flexibility	Societal stakeholders emphasize the importance of individual differences and acknowledge the limitations of brain flexibility
Anticipation	Envisioning the impacts of dominant and alternative future scenarios	Deliberation about envisioned futures and impacts of the neurofeedback (NF) intervention	Three negative impacts were identified by the societal stakeholders:
			1. NF as reductionist method
			2. NF used in future to increase grades
			3. Future misuse of NF as test
Inclusion	Early involvement of diverse stakeholders in deliberation and decision‐making processes	Holding focus groups and interviews with societal stakeholders	Parents considered their inclusion throughout the intervention as necessary
		Discussing ideas on inclusion in focus groups/interviews	Researchers envisioned different role: educating parents rather than involving them
Responsiveness	(Development of a capacity to) respond to the anticipatory and reflective questions generated in inclusive deliberations	Researchers respond to the deliberation results by adapting the intervention (e.g., the framing, content)	Responsive actions by researchers:
			1. Connecting NF experience to person and learning processes, avoiding reductionist interpretation
			2. Framing/focus of the intervention: on developing resilience & wellbeing, instead of maximizing grades
			3. Continued efforts to prevent misuse

## USING RRI IN MBE RESEARCH: A CASE STUDY DEVELOPING A RESPONSIBLE NEUROFEEDBACK‐BASED EDUCATIONAL INTERVENTION

To explore the feasibility and benefits of RRI in MBE research, we conducted a case study on how to responsibly implement a neurofeedback‐based intervention. The aim of the intervention is to convince learners of the plasticity of their own brain and to improve their sense of agency regarding their own learning processes. Such an intervention involves multiple societal stakeholders such as researchers, teachers, parents, and children that each have their own legitimate perspective on the challenges and complexities of the issue. In this section, we first articulate the nature and challenges of the neurofeedback intervention from the perspective of the researchers, and subsequently describe how we applied the RRI framework to investigate these issues from the perspectives of the societal stakeholders. Please note that in the current paper, we will not test the intervention itself, instead we use it as an example of how the RRI framework can be applied to MBE research.

### Nature of the Intervention and Its Challenges as Anticipated by Researchers

Previous research suggests that in addition to differences in cognitive ability, individual differences in one's beliefs about these abilities are also important predictors of school success (Burnette, O'boyle, VanEpps, Pollack, & Finkel, 2013; Costa & Faria, 2018; Dweck & Leggett, 1988; Stankov & Lee, 2017). An important self‐belief is someone's so‐called implicit theory of intelligence (TOI), varying on a continuum between entity beliefs (or fixed mindset), in which someone believes their intelligence and abilities are fixed traits, to incremental beliefs (or growth mindset), in which someone believes their intelligence and abilities are malleable, for example, with effort (Dweck, 1999). Research has shown that TOI influences self‐regulatory behavior, such as goal‐orientation and helpless versus mastery‐oriented strategies, and academic achievement (Burnette et al., 2013). In parallel, mindset interventions have been developed to stimulate a growth mindset in high school students and thereby increase motivation for learning, resilience to setbacks, and educational achievement, with varying success (Sisk, Burgoyne, Sun, Butler, & Macnamara, 2018).

Experience‐dependent plasticity can be seen as a neurobiological basis for the growth mindset. Mindset interventions therefore often contain lessons about brain plasticity, with the aim to make students aware of the brain's capacity to change both structurally and functionally during learning, and that they have influence over this plasticity (e.g., Blackwell, Trzesniewski, & Dweck, 2007). In our current research program, we are developing a mindset intervention including an *experience* of brain plasticity. As previous outcomes of mindset interventions were mixed (Sisk et al., 2018), we aim to generate a stronger effect on student's self‐beliefs by creating an actual experience of their own influence on their neural processes, using electroencephalography‐based neurofeedback. This neurofeedback experience is embedded within a lesson series about plasticity and mindset.

However, as the “plasticity message” may have negative effects in addition to benefits, and our intervention introduces a novel use of neurotechnology (Williamson, 2019), it is important to explore potential negative impacts and risks (e.g., of miscommunication) from the outset of the study. From our researcher's perspective, we mainly foresee potential negative impacts of an emphasis on brain plasticity and ownership of neural learning processes, that is, the plasticity message. The first is that of *increased individual responsibility* (O'Connor et al., 2012): the plasticity message could imply that becoming smarter and successful is your own responsibility. And that if you do not succeed at something, it is because you did not try hard enough. This holds for children as well as parents, as they are also responsible for their child's development. The second risk is the possibility of *unrealistic expectations*: the notion that everyone can become a genius, as long as you try hard enough and receive the right interventions or schooling. This is not realistic, as biological factors are strongly constraining. Although Dweck's work by no means ignores the role of aptitude, the simplified message that reaches teachers and parents is at risk of emphasizing the role of practice and effort too strongly. This overemphasis on malleability is also called the “nurture assumption” (Sokolowski & Ansari, 2018), in which the biological basis of individual differences is largely ignored, leading to inflated expectations of interventions to improve learning ability. Related to this, the third risk is that there are also *misconceptions* that negatively impact the application of mindset interventions and how the mindset message is being used in schools. One misconception about the mindset theory and related research findings is that effort is the holy grail, that we should always praise effort (over talent), independent of the results. However, the correct interpretation of the mindset theory is that it is not merely about effort; the invested effort should result in learning and improving.

### Applying the RRI Framework

The possible negative impacts of the “plasticity message” outlined above, as well as the new use of neuro‐technology central to the intervention (Williamson, 2019), call for deliberation of these impacts on society with society. RRI facilitates the deliberation of possible negative impacts when considering new technological developments, making this neurofeedback intervention a valuable and appropriate case study. When developing a new neurofeedback intervention to be used in schools, the societal stakeholders that are most prominently involved (and affected) are teachers, teenagers, and their parents. Considering the similarities and differences between perspectives of societal stakeholders and researchers, enables us as researchers to broaden our reflection and anticipate future impacts. The RRI study thus aimed to (1) uncover what considerations arise from the perspective of teenagers, parents, and teachers with respect to each dimension of the RRI framework, and (2) how we as researchers could be responsive to these considerations.

#### *Methods*

To ensure that priority is given to the participants' hierarchy of importance, language, and frameworks of understanding, we employed open and flexible qualitative research methods to obtain a view of the societal stakeholders' perspective. The parents, teachers, and researchers participated in semi‐structured interviews. Participation was based on voluntary administration using a self‐selection sampling approach. To ensure the external validity, the different stakeholder groups were aimed to be as diverse as possible. Age, gender, and education level were varied within each group. Five parents (one male and four female, between 30 and 50 years old) and eight teachers (five male and three female, between 24 and 45 years old) participated. Recruitment of both teachers and parents was achieved by messages on social media, flyers, and word of mouth. The researchers that participated were recruited from our own research group (two male and four female, aged between 25 and 40). The teenagers participated in focus group interviews in order to reduce the level of intimidation and give them more opportunity to express themselves freely. Three focus groups were conducted with four to six teenagers per group from both middle and higher‐level education. A total of 16 teenagers was recruited (11 male and five female, between 11 and 14 years old). Recruitment of the teenagers was accomplished via teachers. All participants signed an informed consent before they were subjected to the interview. The teenagers needed their parents' consent before they could participate. All procedures were approved by the local ethics committee of the Vrije Universiteit Amsterdam, Faculty of Behavioural and Movement Sciences. It should be noted that, even though we aimed for as much diversity as possible within de different stakeholder groups, the use of a self‐selection sampling approach may have led to participation bias as opinionated or interested people tend to volunteer more frequently (Robinson, 2014). However, we have no indication that the interviewees had very strong opinions or even particular interest with respect to the neurofeedback intervention. Almost all interviewees were unfamiliar with the term “neurofeedback” before participating in the research. Still, when interpreting the results this consideration should be noted.

The focus groups and interviews were constructed according to a semi‐structured, stepwise design (see Kupper, Krijgsman, Bout, & de Cock Buning, 2007). In the first stage, we asked the interviewees to reflect upon the importance and the flexibility of the brain in the context of learning and education (*reflexivity*). In the second stage, we briefly explained the neurofeedback intervention without emphasizing any applications or drawbacks to limit any bias. Instead, we just outlined the mechanism. We then invited the interviewees to envision possible futures, and contemplating a variety of impacts (*anticipation*). In the third stage, the dimension of *inclusion* was deliberated by enquiring who should be included in the implementation of neurofeedback in school systems and how. See Table 1 for a summary of how the RRI dimensions were implemented during the interviews.

To analyze the transcribed interviews, first, closed coding was used. Within this type of coding, the data is categorized based on pre‐established concepts. The concepts used within this case study were reflexivity, anticipation, and inclusion. Closed coding was followed by open coding, within this type of coding the data is categorized as indicated by the data, no assumptions should be made. Thereby, this type of coding allows for the development of new concepts and categories (Pandit, 1996). To limit researcher bias, multiple coding was conducted during the open coding phase. Subsequently, axial coding was used to uncover the relation between the different concepts and categories (Pandit, 1996). Within the case study the emphasis was mainly on coherencies and discrepancies between the researchers and the societal stakeholder groups, as mismatches between these perspectives could inform the responsive actions. Finally, selective coding was used to integrate the newly developed categories into the initial theoretical framework. Within the case study this signifies the original RRI framework. After each interview a short summary was written and sent back to the interviewee(s) for validation.

#### *Results*

In order to explore the RRI dimension of reflexivity, we analyzed whether the challenges revolving around the “plasticity message,” as predicted by the researchers, were aligned with the perspectives of the societal stakeholders. All interviewees agreed the brain is flexible. Teenager #4 (group 2): “You can always learn new things, for example Chinese” but also stated that there are certain boundaries to this flexibility. Teacher #7: “I think the brain is flexible, but until a certain point. Within your own capacity you can train and improve yourself.” When considering manipulation of the brain to reach maximal capacity almost all interviewees agreed that this maximal capacity differs per teenager. The emphasis should not be on reaching full intelligence but on the individual capacities of the child. Parent #4: “A danger is that parents always want their children to be smarter, but why do we have to be so smart? It is dangerous this emphasis on intelligence. If a child is good with his hands or very social then that is at least as important.”

With respect to the RRI dimension of anticipation, we explored the stakeholders' perspectives on the desirability and potential negative impacts of neurofeedback as a neurotechnological intervention method in schools. Three negative impacts, which were not considered by the researchers (see Nature of the Intervention and Its Challenges as Anticipated by Researchers), were identified. Primarily, neurofeedback was often seen as a reductionist method. Teacher #2: “The learning process is a very complex process and with just a few electrodes on your head you cannot see what these neurons are really doing, so your measurement is already a simplification of what happens in the brain.” Furthermore, human interaction was considered more valuable than the confrontation with brain waves. Parent #1: “A person, teacher will have more influence on the behavior of a child than the confrontation with brainwaves on a screen.” Secondly, the idea that neurofeedback could be used to increase school results received a lot of skepticism from all stakeholders. Teenager #5 (group 1): “Maybe you feel more encouraged before you go to the test, but after the test you become sad again and then next time you are not going to think; 'this will work'.” Moreover, teachers as well as parents felt that increasing results should not be the highest aim. Instead, interventions should be about discovering abilities and talents. Teacher #5: “I think a school is more than a grade factory, you have to help students shape themselves in this important moment in their lives (…).” There appeared to be an adversity toward the achievement‐oriented society. Parent #3: “The aim is growth of your child. Educational level is a metaphor; like we all have the same goal; I think that is not the case. You should make children aware their goal is within themselves.” Finally, the stakeholders foresaw that neurofeedback might be used as some kind of test. Parents as well as teachers seemed irritated by the number of tests teenagers are subjected to these days. They considered the possibility of neurofeedback developing in yet another test as risky and undesirable. Parent #4: “As a means to reach consciousness; there is nothing wrong with that (…) But the moment this information leads to determining something (…) it could lead to stigmatization and medicalization.” They showed little confidence that the use of these new techniques could be controlled. Teacher #1: “But you already know, if these techniques are there they will be used for any application where one sees possibilities.”

The RRI dimension of inclusion considers who should be included and how. This dimension thereby aims to counter top‐down decision‐making. The parents often considered their inclusion as necessary for the intervention to work. Parent #3: “I think it is a combination of different things to induce growth in a child and I don't think it is only about that moment behind the computer but also about how he gives meaning to this. And then it can be good if I as a parent can also talk about this.” The researchers, however, did not consider the role of the parent as prominent within the intervention. Furthermore, the form of inclusion was not agreed upon. Researchers often feel like they have to educate the parents whereas the parents feel they should be included throughout the intervention. Parent #4: “It should not be like; there is the researcher and there are the parents, that [style of interaction] is too frontal. The researcher is on the same level as the child and the parents, not just during the research but also during the interpretation.” Moreover, the parents state that also when evaluating the intervention their involvement is imperative as they are the ones that can deliberate best if their child indeed developed a stronger growth mindset. Parent #2: “The children can say something about awareness, the school about performance but I think also consider the parents. They see the differences in behavior.” In Table 1, the results for each RRI dimension are summarized. The dimension responsiveness will be discussed in section Responsiveness: Adaptations Made to the Intervention Based on the RRI Results.

## DISCUSSION

This case study shows that RRI may well be used as a framework to establish a dialogue with the educational practice and societal stakeholders (e.g., parents) about ethical concerns and desirability of innovations provided by MBE research. We asked the societal stakeholders most prominently involved with the neurofeedback intervention to reflect on what possible effects they anticipated, taking into account their own activities, commitments, and underlying perspectives. This enables us to be responsive to the stakeholders needs and values, by adapting the neurofeedback‐based intervention according to their perspectives (see section Responsiveness: Adaptations Made to the Intervention Based on the RRI Results). These adaptations will ultimately improve its educational and societal value, and reduce the risk of miscommunication.

### Discussion of Main Findings Yielded by the RRI Approach

Interestingly, the negative impacts predicted by us from our researchers' perspective (see section Nature of the Intervention and Its Challenges as Anticipated by Researchers), for example that of unrealistic expectations about brain malleability, did not seem a concern from the societal stakeholder perspective. On the contrary, they emphasize the importance of individual differences and acknowledge the limitations of the brain. They stated that the emphasis should be on developing individual capacities, which are not necessarily related to intelligence. This is a legitimate perspective, but we cannot exclude that there may still be a difference between explicitly formulated opinions, as in our interviews, and more implicit beliefs driving actual behavior. For example, a recent study showed that the self‐reported growth mindset of teachers was not reflected in their teaching behavior, that is, it was not associated with providing more growth‐oriented feedback to students (de Kraker‐Pauw, Van Wesel, Krabbendam, & Van Atteveldt, 2017). In other words, although stakeholders may explicitly acknowledge the importance of individual differences, they may still behave differently. Therefore, this researcher's concern may still be of importance. It is important to emphasize that the difference between explicit opinions versus implicit beliefs and behavior does not make the stakeholders' concern less legitimate, but rather less easy to solve. The challenge for future RRI approaches within MBE research therefore is to design deliberative processes in such a way that these kinds of ambiguities are addressed.

Another interesting result was that the societal stakeholders anticipated negative impacts we as researchers did not predict. For example, the neurofeedback‐based intervention was seen as a reductionist method with too much emphasis on increasing learning achievements, with the risk of developing into yet another test. This is partly a concern about the possible normative nature of the intervention, related to the concern above. The fear that the neurofeedback technology would lead to reductionism echoes what is reported in previous neuro‐ethical studies which have also emphasized the potential reductionist nature of neuroimaging (Edelenbosch, Kupper, & Broerse, 2015; Racine, Bar‐Ilan, & Illes, 2005). This underlines the capability of societal stakeholders to reflect on technological innovations, but we would also like to note that these deliberations are also socially and politically constructed (see Latour & Woolgar, 1979). We need the social stakeholders to give meaning to these constructs. Finally, we found that the parents felt they should be included during the execution as well as evaluation of the intervention. The parents make an interesting point arguing that, if a stronger growth mindset and more resilience to setbacks are the objectives of the intervention, they should be included throughout the intervention to yield sustainable effects.

### Responsiveness: Adaptations Made to the Intervention Based on the RRI Results

The insights uncovered by applying the RRI‐framework of Stilgoe et al. (2013) to our research, helped us researchers in developing and implementing the intervention in such a way that we will achieve the right impacts. In other words, we are responsive to the stakeholder's input, in the following ways (a summary of our responsive actions is provided in Table 1). First of all, we included meaningful connections between a teenager's “brain waves” to the person and his or her learning processes, to avoid that the intervention leads to reductionism of children to their brains or their brain waves (see also Edelenbosch, Kupper, & Broerse, 2015). The final intervention comprises four sessions, and only in one session the teenagers are provided with the neurofeedback experience. The other three sessions are devoted to explain learning and plasticity in the brain, the consequences of thinking and acting from different mindsets, and connecting this all to the neurofeedback experience and school situations. By this careful embedding of the neurofeedback experience, we explicitly emphasized other dimensions of a person's identity and learning processes, and thereby minimize the risk of reducing the teenagers to their brains or brain waves. Moreover, as it was mentioned that human interaction was considered more valuable than the “confrontation with brain waves,” we included many social exchanges in the different sessions, with peers as well as the teacher. For example, during the second session with the subject “mindsets,” teenagers gave each other feedback and tips on how to react with a growth mindset when confronted with a challenging situation in school. In the last session, teenagers discussed the neurofeedback session of the week before together with the teacher, to stimulate dialogue to ensure that connections to the person and learning processes were made.

Secondly, we focused the intervention on developing resilience and wellbeing in children, rather than putting too much focus on maximizing grades and intelligence. Importantly, this is also how we communicate about the intervention with the schools and teachers, and the participating teenagers. The focus on wellbeing and resilience, rather than maximizing grades and intelligence, was effectuated by the examples and exercises we chose. For example, in the first session on brain plasticity, we used examples of nonacademic learning, such as mirror drawing and juggling. In the second session (on mindset), we emphasized that mindset can vary between subjects at school (math, sports, biology). In addition, we showed a video about perseverance and failure as part of learning, with many sports examples. Finally, for all assignments, teenagers were free to choose their own subjects or situations in which they experienced challenge or struggles, rather than reflecting on general intelligence. In response to the point that the focus should not strongly be on “increasing results,” the last session of the intervention ends with an assignment, in which the teenagers send an actual postcard to their future selves. We asked them to write to themselves how a growth mindset would be useful for them in the next year.

Thirdly, we anticipate on future misuse of our intervention (e.g., commercial, see Singh & Rose, 2009), for example, by keeping the intellectual property rights to control future applications. Although we hold these specific rights, similar kinds of technology and applications can and will likely be developed with increasing ease, facilitated by for example, low‐cost headsets and open‐source software. We will make continued efforts to share the RRI methodology and our values on this topic, in the form of the current article, scientific presentations, and societal interactions.

Finally, related to the deliberations on *inclusion*, from a feasibility standpoint, we could not include the parents in the execution of the full intervention study. For example, because of the randomized controlled trial nature with future posttests, we cannot unblind the conditions yet, making it very hard to involve parents. It should be mentioned, however, that including parents as stakeholders during the development of the intervention was very useful, and could be an alternative when it is not feasible to have them participate in the execution of the intervention itself. Regarding the desire of parents to be included in the evaluation of the intervention, we will more actively think how we can include them in the final evaluation stage, to supplement the intervention effects with parent's observations.

### General Implications and Conclusions

The abovementioned responsive actions are specific to our case study and therefore may have limited generalizable value. However, the aim of the case study was to test the feasibility of RRI within MBE research. We conclude that in our case study, the RRI framework was fundamental in uncovering the societal stakeholders needs and values. Just anticipating possible negative effects from the perspective of us researchers is not enough to conduct responsible research. The societal stakeholders need to be involved at an early stage as their perspectives are valuable and not necessarily align with the perspectives of us researchers. The case study does not only show that societal stakeholders are capable but also needed to deliberate technical, social, and political constructs of innovations regarding neuroscience applications in a school setting.

The responsive change integrated in the RRI framework enhances relationships in and with the community in which one does research. This responsiveness is especially relevant in MBE research as there is a strong link between cognitive and developmental neuroscientific research and education, and only by being responsive to stakeholders' perspectives this link may become the two‐way dialogue it needs to be (Ansari et al., 2011). To conclude, the RRI framework offers a systematic and feasible means to establish such a dialogue and guide MBE research toward the right educational and societal impacts. RRI may be helpful for MBE researchers to fulfill the need for public dialogue efficiently and systematically, to avoid miscommunications, and optimize alignment with values and needs in the educational practice.
